# Supplementary material for: Cell Heterogeneity Analysis Revealed the Key Role of Fibroblasts in the Magnum Regression of Ducks
Source: Animals (Basel). 2024 Apr 1;14(7):1072. doi: 10.3390/ani14071072 (PMC11011120; doi:10.3390/ani14071072)
Supplement: Supplementary file 1 [file animals-14-01072-s001.zip › Supplementary Figure S3.pdf]

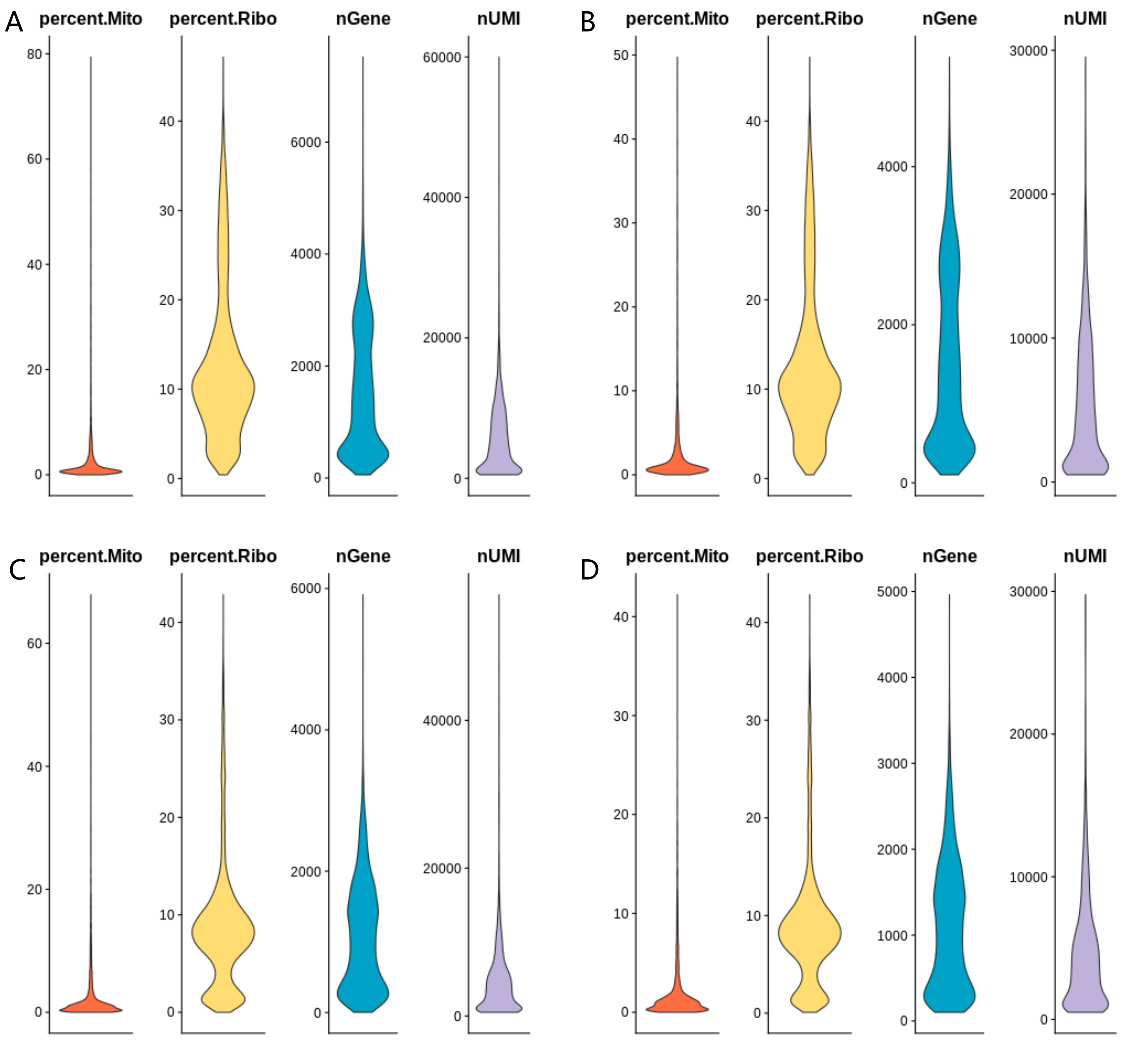


**Figure S3.** The distribution of basic information of cells in each sample before and after the filter. **A** The distribution of basic information of cells in O_C before the filter; **B** The distribution of basic information of cells in O_C after the filter; **C** The distribution of basic information of cells in O_L before the filter; **D** The distribution of basic information of cells in O_L after the filter; The percent. Mito shows the distribution of mitochondrial gene expression level of cells in each sample; The percent. Ribo shows the distribution of ribosome gene expression level of cells in each sample; The nGene shows the distribution of gene number of cells in each sample; The nUMI shows the distribution of UMI number of cells in each sample; O_C: magnum of ceased-laying duck; O_L: magnum of laying duck.
