# Supplementary material for: Cell Heterogeneity Analysis Revealed the Key Role of Fibroblasts in the Magnum Regression of Ducks
Source: Animals (Basel). 2024 Apr 1;14(7):1072. doi: 10.3390/ani14071072 (PMC11011120; doi:10.3390/ani14071072)
Supplement: Supplementary file 1 [file animals-14-01072-s001.zip › Supplementary Figure S2.pdf]

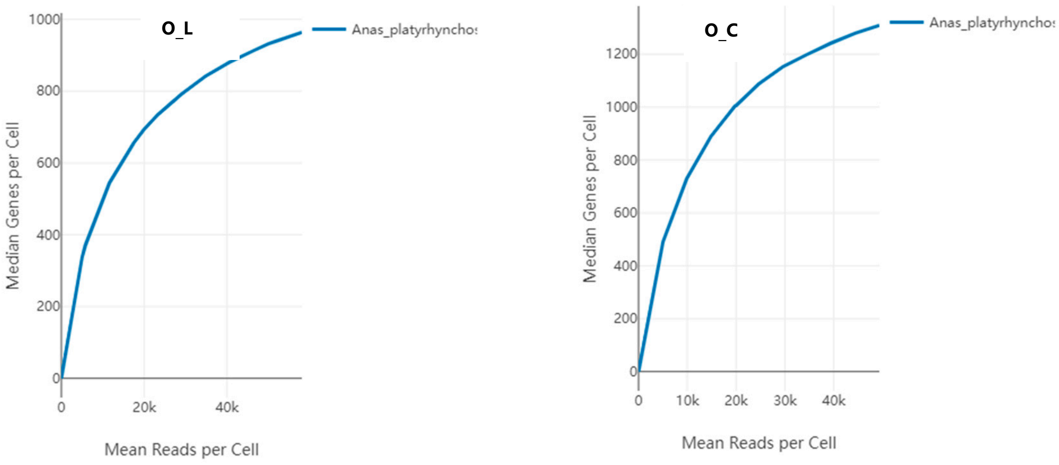

Figure S2. Median number of genes detected per cell as a function of mean reads per cell. O_C: magnum of ceased-laying duck; O_L: magnum of laying duck.
