# Supplementary material for: Cell Heterogeneity Analysis Revealed the Key Role of Fibroblasts in the Magnum Regression of Ducks
Source: Animals (Basel). 2024 Apr 1;14(7):1072. doi: 10.3390/ani14071072 (PMC11011120; doi:10.3390/ani14071072)
Supplement: Supplementary file 1 [file animals-14-01072-s001.zip › Supplementary Table S1.pdf]

Table. S1. Statistical results of the sequencing data of magnum samples in different laying status.

Sample	Number of Reads	Valid Barcodes	Sequencing Saturation	Q30 Bases in Barcode	Q30 Bases in RNA Read	Q30 Bases in UMI
O_C	385,449,534	95.4%	67.6%	95.9%	93.7%	94.0%
O_L	342,320,624	93.0%	73.6%	96.1%	94.4%	94.2%

O_C: magnum of ceased-laying duck; O_L: magnum of laying duck; Number of Reads: the number of reads obtained from each sample; Valid Barcodes: the ratio of reads with valid barcode to all the reads, each valid barcode represented one cell; Q30 Bases in Barcodes: the percentage of bases in barcode whose mass fraction is greater than or equal to 30; Q30 Bases in RNA Read: the percentage of bases in RNA read whose mass fraction is greater than or equal to 30; Q30 Bases in UMI: the percentage of bases in UMI whose mass fraction is greater than or equal to 30.
