# Supplementary material for: Cell Heterogeneity Analysis Revealed the Key Role of Fibroblasts in the Magnum Regression of Ducks
Source: Animals (Basel). 2024 Apr 1;14(7):1072. doi: 10.3390/ani14071072 (PMC11011120; doi:10.3390/ani14071072)
Supplement: Supplementary file 1 [file animals-14-01072-s001.zip › Supplementary Table S2.pdf]

Table. S2. Statistical results of quality by Cell Ranger of magnum samples in different laying status.

Sample	ENC	MRC	MGC	RMG	RMCG	RMC (Integenic)	RMC (Intronic)	RMC (Exonic)	RMCT	RMAG	FRC	TGD
O_C	7,811	49,347	1,309	71.7%	65.2%	4.7%	12.1%	48.3%	46.2%	1.0%	78.4%	19,490
O_L	5,897	58,049	964	76.5%	68.9%	8.5%	6.7%	53.7%	52.1%	0.7%	65.5%	18,270

O_C: magnum of ceased-laying duck; O_L: magnum of laying duck; ENC: estimated number of cells; MRC: mean reads per cell; MGC: median genes per cell; RMG: reads mapped to genome; RMCG: reads mapped confidently to genome; RMC (Integenic): reads mapped confidently to intergenic regions; RMC (Intronic): reads mapped confidently to intronic regions; RMC (Exonic): reads mapped confidently to exonic regions; RMCT: reads mapped confidently to transcriptome; RMAG: reads mapped antisense to gene; FRC: fraction reads in cells; TGD: total genes detected.
