# Supplementary material for: Cell Heterogeneity Analysis Revealed the Key Role of Fibroblasts in the Magnum Regression of Ducks
Source: Animals (Basel). 2024 Apr 1;14(7):1072. doi: 10.3390/ani14071072 (PMC11011120; doi:10.3390/ani14071072)
Supplement: Supplementary file 1 [file animals-14-01072-s001.zip › Supplementary Table S3.pdf]

Table S3. Statistical table of cell filtration.

Sample	before_filter_cell_num	after_filter_cell_num	precent
O_C	7,811	7,319	93.70%
O_L	5,897	5,113	86.71%

O_C: magnum of ceased-laying duck; O_L: magnum of laying duck.
